# SmoothT—a server constructing low-energy pathways from conformational ensembles for interactive visualization and enhanced sampling

**DOI:** 10.1093/bioinformatics/btad176

**Published:** 2023-04-05

**Authors:** René Staritzbichler, Nikola Ristic, Tülin Stapke, Peter W Hildebrand

**Affiliations:** Institute of Medical Physics and Biophysics, University of Leipzig, Leipzig 04107, Germany; Institute of Medical Physics and Biophysics, University of Leipzig, Leipzig 04107, Germany; Institute of Medical Physics and Biophysics, University of Leipzig, Leipzig 04107, Germany; Institute of Medical Physics and Biophysics, University of Leipzig, Leipzig 04107, Germany; Institute of Medical Physics and Biophysics, Charité Universitätsmedizin Berlin, Corporate Member of Freie Universität Berlin und Humboldt- Universität zu Berlin, Berlin 10117, Germany; Berlin Institute of Health at Charité-Universitätsmedizin, Berlin 10178, Germany

## Abstract

**Motivation:**

The SmoothT software and webservice offers the construction of pathways from an ensemble of conformations. The user provides an archive of molecule conformations in Protein Databank (PDB) format, from which a starting and a final conformation need to be selected. The individual PDB files need to contain an energy value or score, estimating the quality of the respective confirmation. Additionally, the user has to provide a root-mean-square deviation (RMSD) cut-off, below which conformations are considered neighboring. From this, SmoothT constructs a graph that connects similar conformations.

**Results:**

SmoothT returns the energetically most favorable pathway within in this graph. This pathway is directly displayed as interactive animation using the NGL viewer. Simultaneously, the energy along the pathway is plotted, highlighting the conformation that is currently displayed in the 3D window.

**Availability and implementation:**

SmoothT is available as webservice at: http://proteinformatics.org/smoothT. Examples, a tutorial, and FAQs can be found there. Ensembles up to 2 GB (compressed) can be uploaded. Results will be stored for 5 days. The server is completely free and requires no registration. The C++ source code is available at: https://github.com/starbeachlab/smoothT.

## 1 Introduction

Biological processes commonly involve transitions between distinct molecular states, which in most cases are related to conformational changes. Examples of such pairs of states are bound versus unbound, unfolded versus folded, and inactive versus active. Driving force of molecular processes is generally the aim of these systems to reach a minimum in free energy. The correct path between starting and final state is expected to be the one with the lowest energy barrier. Determining the most realistic path means searching through the energy landscape for the one with the lowest energy (Smidstrup et al. 2014).

Mapping complete energy landscapes of macro-molecules will certainly remain unattainable in the near future due to their immense complexity. The most common modeling techniques for macro-molecules are molecular dynamics (MD) and Monte Carlo (MC). While in general MD calculates the time-evolution of the system, following Newton’s classical equation of motion, MC approaches create random samples following a given distribution, typically the Boltzmann distribution. Hence, MC algorithms are successfully applied whenever the size or complexity of the search space prohibits both a systematic scan or MD, since MD would simply spend too much time sampling local minima of the free-energy landscape—although methods exist to enhance the sampling rate of MD, e.g. umbrella sampling (Torrie and Valleau 1977) or meta-dynamics (Laio and Parrinello 2002).

SmoothT, on the other hand, is constructing pathways from previously computed ensembles of macro-molecular conformations. It therefore allows to combine results from different techniques and provides an efficient way to create starting points for enhanced sampling techniques with little initial assumption about the pathway. It should be noted, however, that other methods generally aim to recover transition pathways by taking the sampling itself into account, yielding pathways as they would have occurred according to the underlying physical model (Metzner et al. 2009; Schütte et al. 2011; Bowman et al. 2014), thus providing conformational and kinetic information about the pathway.

## 2 Materials and methods

**Figure 1:**
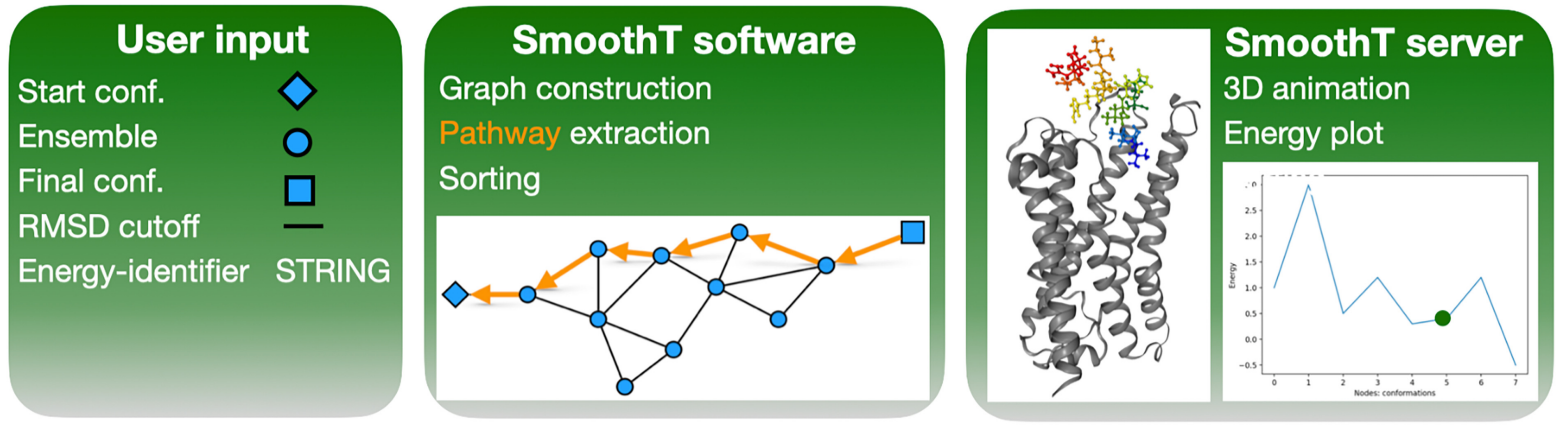
The user provides the conformational ensemble, the starting and ending conformation, the RMSD threshold, and an identifier for the energy values in the PDB files. SmoothT then constructs a graph, connecting similar conformations in a backtrace. SmoothT extracts the most favorable pathway according to two energy-related criteria. The server displays an animation of the transition and an energy plot (right box). The big dot in the plot indicates the energy of the current snapshot

The path determination in SmoothT is performed in two phases. First, a graph is constructed containing all putative pathways. Second, the most favorable pathway is selected. The graph is constructed by calculating the similarity between conformations. Conformations that are below the user-defined RMSD cut-off are connected by an edge. The RMSD is calculated without superimposition. Nodes contain the conformation with the associated energy. The energies are shifted by the lowest energy found in the graph (see the method section on the server for illustrations). The most favorable pathway is determined in a dynamic programming approach, in which for each node it is decided locally which of its connected nodes leads to the assembly of the best sub-path. The selection follows two criteria. The first is the energy barrier, the maximum energy along the path. The second is the area under the RMSD-energy curve. The integral over the energy, shifted by the minimum energy, will favor first short pathways and for pathways of similar length, it will favor those with an overall lower energy. The actual pathway is constructed by a back-propagation. The server displays both the trajectory using the NGL viewer (Rose and Hildebrand 2015) as well as an energy diagram, highlighting the energy of the snapshot currently displayed in the NGL window (see Figure 1).

## 3 Examples

One example of usage would take snapshots from several independent MD simulations to connect them into a simple representation of the simulated transition. Another example would use an ensemble of Monte Carlo docking poses as input and connect them into a simple estimation of the transition from the unbound to bound state. The output pathways themselves can serve as starting point of simulations, e.g. in an enhanced sampling approach. In this way, SmoothT provides a natural link between the global sampling of MC methods and the local sampling of MD simulations. SmoothT has no restrictions on the types and number of molecules that can be used.
